# Membrane-associated RING-CH 7 inhibits stem-like capacities of bladder cancer cells by interacting with nucleotide-binding oligomerization domain containing 1

**DOI:** 10.1186/s13578-024-01210-y

**Published:** 2024-03-10

**Authors:** Junlong Zhuang, Lingli Zhang, Siyuan Zhang, Zhongqing Zhang, Tianlei Xie, Wei Zhao, Yantao Liu

**Affiliations:** 1 Department of Urology, Nanjing Drum Tower Hospital, Affiliated Hospital of Medical School, Nanjing University, Nanjing, China; 2 Nanjing Drum Tower Hospital Clinical College of Nanjing Medical University, Nanjing, China; 3 Institute of Urology, Nanjing University, Nanjing, China; 4 Department of Pharmacy, West China Second University Hospital, Sichuan University, Chengdu, China; 5 Evidence-Based Pharmacy Center, West China Second University Hospital, Sichuan University, Chengdu, China; 6 Key Laboratory of Birth Defects and Related Diseases of Women and Children, Ministry of Education, Sichuan University, Chengdu, China; 7 School of Laboratory Medicine, Chengdu Medical College, Chengdu, China; 8 Clinical Laboratory, Clinical Medical College and The First Affiliated Hospital of Chengdu Medical College, Chengdu, China

**Keywords:** Bladder cancer, Cancer stem-like cells, MARCH7, NOD1, Ubiquitination

## Abstract

**Background:**

Cancer stem-like capacities are major factors contributing to unfavorable prognosis. However, the associated molecular mechanisms underlying cancer stem-like cells (CSCs) maintain remain unclear. This study aimed to investigate the role of the ubiquitin E3 ligase membrane-associated RING-CH 7 (MARCH7) in bladder cancer cell CSCs.

**Methods:**

Male BALB/c nude mice aged 4–5 weeks were utilized to generate bladder xenograft model. The expression levels of MARCHs were checked in online databases and our collected bladder tumors by quantitative real-time PCR (q-PCR) and immunohistochemistry (IHC). Next, we evaluated the stem-like capacities of bladder cancer cells with knockdown or overexpression of MARCH7 by assessing their spheroid-forming ability and spheroid size. Additionally, we conducted proliferation, colony formation, and transwell assays to validate the effects of MARCH7 on bladder cancer CSCs. The detailed molecular mechanism of MARCH7/NOD1 was validated by immunoprecipitation, dual luciferase, and in vitro ubiquitination assays. Co-immunoprecipitation experiments revealed that nucleotide-binding oligomerization domain-containing 1 (NOD1) is a substrate of MARCH7.

**Results:**

We found that MARCH7 interacts with NOD1, leading to the ubiquitin–proteasome degradation of NOD1. Furthermore, our data suggest that NOD1 significantly enhances stem-like capacities such as proliferation and invasion abilities. The overexpressed MARCH7 counteracts the effects of NOD1 on bladder cancer CSCs in both in vivo and in vitro models.

**Conclusion:**

Our findings indicate that MARCH7 functions as a tumor suppressor and inhibits the stem-like capacities of bladder tumor cells by promoting the ubiquitin–proteasome degradation of NOD1. Targeting the MARCH7/NOD1 pathway could be a promising therapeutic strategy for bladder cancer patients.

**Supplementary Information:**

The online version contains supplementary material available at 10.1186/s13578-024-01210-y.

## Introduction

Bladder cancer is a prevalent malignant tumor that poses a significant threat to human health [1]. It ranks among the top 10 most frequently diagnosed cancers worldwide, with approximately 573,000 new cases and 213,000 deaths reported [2]. Despite advances in diagnostic and therapeutic strategies, the clinical outcomes of bladder cancer are still far from satisfactory [3]. Therefore, it is imperative to develop effective approaches for bladder cancer prevention and targeted therapy. Recent evidence has highlighted the crucial role of cancer cell stemness and stem-like capacities in bladder cancer development [4, 5]. Consequently, understanding the molecular mechanism underlying cancer stem-like cells in bladder cancer cells holds great importance.

Bladder cancer stem-like cells (CSCs), characterized by higher tumorigenicity, invasion ability, treatment-resistant, represent an ideal target for therapy [4, 6, 7]. Emerging data characterized some important molecules and signal pathways in bladder CSCs. Hua Wei reported that IL6/IL6R/STAT3 axis is critical for the maintain of the stem-like properties, and blockage of IL6R inhibit the invasion, migration and tumorigenicity of bladder cancer CSCs [8]. Previous study indicated targeting the autocrine regulatory loop, YAP/TEAD1/PDGF-BB/PDGFR in CSCs abrogates bladder tumor initiation and chemotherapy resistance [7]. Isorhapontigenin (ISO) was reported to decrease CD44 expression in mRNA level via downregulation of transcriptional factor, SP1, and protein level by decrease USP28 expression, which reduced CD44 protein stability [6]. The ISO attenuated CD44 expression results inhibition of stem cell-like properties and invasion of bladder cancer cells. Toshihiko Torigoe et al. identified the glutamate receptor, ionotropic, kainite 2 (GRIK2)-derived antigenic peptide was specifically expressed in CSCs. The cytotoxic T lymphocytes which recognize GRIK2 specifically, overcome bladder cancer cells treatment-resistance [9]. However, these present finding do not fully elucidate the molecular mechanism underlying CSCs of bladder cancer.

Several stem cell markers have been identified in bladder cancer [10–12]. CD44 is widely used as a bladder cancer stem cells biomarker [13], and interference of CD44 expression inhibits stemness and progression in bladder cancer cells [5, 14]. CD133 + cells have certain characteristics of bladder cancer stem cells [15], and the expression of CD133 in tumors has been significantly correlated with urothelial carcinoma grade and stage [16]. Additionally, different stem-like markers and drug-resistant genes, such as OCT-4, SOX2 and ATP Binding Cassette Subfamily G Member 2 (ABCG2), are closely related to spheroid-forming ability and impact bladder tumor formation [17–19].

The membrane-associated RING-CH (MARCH) family comprises MARCH1 to 11. Members of the MARCH family exhibit diversity and participate in multiple physiological functions, including sperm formation, membrane transfer, and lipid synthesis [20]. Emerging evidence suggests that MARCH family proteins are involved in tumor progression [21]. Overexpression of MARCH1 promotes ovarian cancer progression through the regulation of NF-κB and Wnt/β-catenin signaling pathways [22] while MARCH1 overexpression in oral squamous cell carcinoma promotes tumor progression through regulation of the PH domain and leucine-rich repeat protein phosphatase (PHLPP) 2 [23]. Moreover, MARCH2/6/8 is highly expressed in colon cancer [24], papillary thyroid cancer [25], esophageal cancer [26], accelerated cancer progression. Previously, MARCH7 was reported to be stabilized by Ubiquitin Specific Peptidase 9 X-Linked (USP9X) and Ubiquitin Specific Peptidase 7 (USP7), which deubiquitylate MARCH7 in the cytosol and nucleus, respectively [27]. Additionally, the MALAT1/MARCH7 Autophagy Related 7 (ATG7) feedback loop plays a critical role in promoting autophagy, migration, and invasion in ovarian cancer [28]. Furthermore, MARCH7 is involved in snail-mediated pathways and facilitates endometrial cancer metastasis [29]. However, the involvement of MARCH7 in bladder cancer progression and its potential mechanisms remain unclear.

Nucleotide-binding oligomerization domain 1 (NOD1) has been reported to be involved in innate immunity, acting as a pattern-recognition receptor (PRR) that binds to bacterial peptidoglycans and initiates inflammation [30, 31]. In 2006, da Silva Correia et al. reported that NOD1 inhibited estrogen-dependent breast tumor growth [32]. NOD1 also abrogates hepatocellular carcinoma proliferation and enhances the tumor response to chemotherapy [33]. In contrast, NOD1 promotes colorectal carcinogenesis [34] and colon cancer metastasis [35]. Additionally, NOD1 accelerates tumorigenesis, tumor metastasis, and NOD2 expression in human squamous cervical cancer [36]. Although NOD1 gene polymorphisms are associated with an altered risk of bladder cancer [37], the precise role of NOD1 in bladder cancer and its detailed molecular mechanisms remain unclear.

In this study, we aimed to elucidate the molecular events involved in bladder cancer stemness, aiming to provide a theoretical basis for the diagnosis and treatment of bladder cancer.

## Materials and methods

### Bladder cancer tissue and cells

Clinicopathological data and tissue specimens were obtained from 30 patients who underwent surgical resection for bladder cancer at the Department of Urology, Nanjing Drum Tower Hospital from September 2019 to September 2020. All patients were confirmed to have bladder cancer through pathological examination, and they had not received any prior radiotherapy, chemotherapy, or other cancer-related treatments. Written informed consent was obtained from all patients, and the study was approved by the ethics committee of Nanjing Drum Tower Hospital.

Human bladder cancer cell lines (T24, 253J, J82, 5637, and RT-4) and human normal bladder epithelial cells (SV-HUC-1) were obtained from the ATCC Culture Collection. The cells were cultured in 1640 medium (Thermo Fisher Scientific) supplemented with 10% FBS (Thermo Fisher Scientific), 100 IU/mL penicillin, and 100 μg/mL streptomycin (Beyotime, Shanghai, China). The cells were maintained in a cell incubator (Thermo) at 37 °C with 5% CO_2_.

### Isolation and culture of cancer stem-like cells

The side population cells, representing the cancer stem cells, were isolated from bladder cancer cell lines (T24 and 253J) using the ALDEFLUOR^®^ assay (Stemcell Technologies, Vancouver, BC, Canada), as previously described [38, 39]. The sorted side population cells were confirmed to possess cancer stem-like cell properties by tumorigenesis in vivo and in vitro, as well as OCT4 expression detection (Additional file 1: Fig. S1). The isolated cancer stem cells were cultured in DMEM/F12 medium supplemented with 2% B27, 40 ng/mL basic fibroblast growth factor (bFGF), and 20 ng/mL epidermal growth factor (EGF).

### Plasmid construction

Wild-type MARCH7 (MARCH7-WT), mutant MARCH7 (MARCH7-Mut), and NOD1 were amplified using Premix-PrimeSTAR-HS (TAKARA, Dalian, China). The DNA fragments were extracted using a MiniBEST Agarose Gel DNA Extraction Kit Ver.4.0 (TAKARA) and cloned into a pcDNA3.1 (+) empty vector using BamHI and XhoI enzymes (Thermo Fisher Scientific). The MARCH7 (NM_001282805.2) and NOD1 (NM_006092.4) sequences were obtained from the National Center for Biotechnology Information (NCBI). The MARCH7-WT and MARCH7-Mut constructs were tagged with a Flag tag. The plasmids were designed and constructed by Shanghai Yuanmu Biotechnology Co., Ltd. (Shanghai, China). The plasmids were used for stable transfection of cancer cells.

### Cell transfection and construction of stable transfected cell lines

The cancer cells were seeded in 6-well plates at a density of 2 × 10^6^ cells per well. When the cells reached 70% confluence, the culture medium was aspirated, and 1 μg MARCH7-pcDNA3.1 or pcDNA3.1 (empty vector) were mixed with DMEM, incubated for 5 min, mixed with LipofectamineTM2000 (Invitrogen), and incubated for 20 min. The mixture (400 μl) was added to the cell medium, and 1 ml of cell culture medium was added after 6 h. The cells were further cultured for 1 day, added G418 (Sigma) into medium with 800 μg/ml concentration. After 30 days selection, the transfected stem cells with MARCH7-vector and the empty vector were referred to as the MARCH7 group and the vector group, respectively.

Same as above procedure, cells were transfected with different concentrations (1 and 1.5 μg) of Flag-MARCH7-WT or MARCH7-Mut, the transfection efficiency was determined by western blotting. Neomycin was added to the screen to ensure stable transfection. The proteasome inhibitor, MG132 (1 µM), was added to the control and experimental groups. After 10 h, the cells were washed. After trypsinization, the cells were centrifuged at 800 rpm for 5 min, and the complete medium was added. The cells were resuspended in a cryopreservation tube placed in a − 80 °C freezer overnight. Flag-MARCH7-WT and Flag-MARCH-Mut were constructed to verify the binding of MARCH7 and NOD1.

### Spheroid formation assay

The effect of MARCH7 on spheroid formation was investigated in bladder cancer stem cells. The cells were seeded in ultra-low attachment 96-well plates (Corning, NY, USA) at a density of 200 cells per well. The plates were gently swirled to distribute the cells evenly and incubated for 7 days to form spheroids. The number and size of spheroids were observed and measured under a microscope.

### Bioinformatics analysis

The indicated genes expression were analyzed by “Gene Differential Express” in the “Pan-Cancer” module of the STARBASE [40] (http://starbase.sysu.edu.cn/index.php). “MARCH7” was entered in the “Gene” option, and MARCH7 expression data for various cancers was obtained. Bladder urothelial carcinoma (BLCA) was selected to obtain mRNA expression data of MARCH7 mRNA in bladder cancer tissue. mRNA levels were obtained from RNA-seq data using the log2 algorithm (FPKM + 0.01). Additionally, we used UbiBroswer online tool (http://ubibrowser.bio-it.cn/ubibrowser/) to predict the potential substrates of MARCH7 following the instructions [41]. Afterwards, we detected the top 10 substrates of MARCH7 by conducting co-IP and western blot.

### Quantitative real-time PCR (q-PCR)

The levels of MARCH family genes were detected using q-PCR. Total RNA was separated from tissues frozen in liquid nitrogen, as well as from bladder cancer cells and normal bladder epithelial cells, by lysis with TRIzol reagent. After the RNA concentration was determined, the total RNA was reverse-transcribed into cDNA. Using cDNA as a template, qPCR amplification was performed using the SYBR Premix Ex TaqTM qRT-PCR kit (TaKaRa, Dalian, China). The primers specific for MARCH family genes are listed in Table 1 and were purchased from Ribobio. (Gungzhou, China). The reaction conditions included pre-denaturation at 95 °C for 5 s, denaturation at 95 °C for 5 s, and annealing at 60 °C for 30 s, total 40 cycles. Using GAPDH as an internal reference, the relative expression of different genes in MARCH7-transfected and vector-transfected cells was computed using the 2^−ΔΔCt^ method.

**Table 1:** Primers for q-PCR

Genes	Forward (5′–3′)	Reverse (5′–3′)
MARCH1	CAC TGC AAC TGT TGT GTC CG	TGG GGT ACA TTT CCC GTG AG
MARCH2	TTG GAC ACA CCG AGT GAT GG	AAG CCA CTT CTC CAG ACA GC
MARCH3	CGT GAG CAG CCA CCG ACT	CAT ACA GGA TTT CCA TCG TCC TCG
MARCH4	ACG TGG TGT GCA TAG GTC TC	TGC CTC CTG CCT TTT GAT CC
MARCH5	CAG ATG CTG GAC AGA AGT TGC	TTC ATC CAC CCA GCG TTG TA
MARCH6	ACA CCG CGG AGG AAG ACA TA	CGA CTG TGT TTC AGC CAT TGA
MARCH7	TGC ATT TCC TCA GTC ACG GG	CCA GGG CAG AAA GCA TTC AA
MARCH8	GCC CCA TGG AGT TTG TCA TC	CAT GGA TTT CAA GTG CGG AGC
MARCH9	CAT CAG CCC TGC CTC ATC C	GAT GGC CTG CCA CTG C
MARCH10	CAG AGC CGA CTT GGT GGA AT	TTG GTG CTT GGT CTG CTT CA
MARCH11	CTT CAG CCC CTA TGC AGT GT	CCT TCA TGA ACA ATG AGA CCT ATG C

### Immunoblotting and immunohistochemistry (IHC)

Protein expression levels of MARCH7 and NOD1 were detected using immunoblotting. Cells were lysed with RIPA buffer (Beyotime) supplemented with protease and phosphatase inhibitors. Protein concentrations were determined using a BCA Protein Assay Kit (Beyotime). Protein samples were separated by SDS-PAGE and transferred to PVDF membranes (Bio-Rad). The membranes were incubated with primary antibodies against -MARCH7 (Abcam, Cambridge, UK), NOD1 (Abcam), β-actin (Abcam), OCT4 (Abcam), CD44 (Thermo Fisher), CD133 (Thermo Fisher), PCNA (Novus Biologicals, Colorado, USA),Cyclin A1 (Novus Biologicals), N-cadherin (Novus Biologicals), E-cadherin (Novus Biologicals), Tubulin-α (Novus Biologicals), Flag (Novus Biologicals), HA (Novus Biologicals), NOD2 (Novus Biologicals), P65 (Novus Biologicals). They were followed by secondary antibodies conjugated to horseradish peroxidase (HRP) (Abcam). Protein bands were visualized using enhanced chemiluminescence (ECL) reagents (Millipore, Billerica, MA, USA). Protein bands were visualized using enhanced chemiluminescence (ECL) reagents (Millipore, Billerica, MA, USA). Band intensities were quantified using ImageJ software.

The expression of MARCH7 and NOD1 in bladder tumors and adjacent normal tissues was examined using IHC staining [42]. Paraffin-embedded tissue sections were deparaffinized and rehydrated. Antigen retrieval was performed by heating the sections in citrate buffer (pH 6.0) using a microwave. The sections were incubated with primary antibodies against MARCH7 (Abcam) and NOD1 (Abcam), followed by HRP-conjugated secondary antibodies (Abcam). The staining was visualized using 3,3′-diaminobenzidine (DAB) substrate (Beyotime). The sections were counterstained with hematoxylin, dehydrated, and mounted.

### Flowing cytometry detecting CSCs marker CD44 and CD133

The stable transfected T24^*CSCs*^ and 253 J ^*CSCs*^ were collected by centrifuging at 100*g* × 5 min. Then, they were washed with PBS gently and centrifuged for three times. Next, they were suspended anti-CD44-FITC- or anti-CD133-PE-contained buffer (140 mM NaCl, 2.5 mM CaCl_2_, 10 mM HEPES, pH7.2) for 30 min. Finally, the stained cells were objected to flowing cytometry analysis. The obtained data were analyzed and showed by using the Flow Jo V10 (Tree Star, California, Auckland, Fairbert Street, USA).

### Cell viability assay

Cell viability was assessed using a Cell Counting Kit-8 (CCK-8) assay (Dojindo, Kumamoto, Japan). The transfected cells were seeded in 96-well plates at a density of 5000 cells per well and incubated for 24, 48, 72, and 96 h. CCK-8 solution (10 μl) was added to each well, and the plates were incubated for 2 h at 37 °C. The absorbance at 450 nm was measured using a microplate reader (BioTek, Winooski, VT, USA).

### Colony formation assay

Stem-like cells were seeded in 6-well plates at a density of 300 stably transfected cells per well. The medium was changed every two days. After incubation for 14 d, the colonies were stained with crystal violet. Briefly, the medium was aspirated, 1 ml methanol was added to each well, and the cells were fixed for 30 min. Then, methanol was discarded, and 0.5% crystal violet solution (Beyotime) for 1 h at room. Finally, the colonies were gently rinsed with crystal violet solution, dried, and the number of colonies (≥ 50 cells) was counted under a microscope (Olympus Corporation). All operations require caution to prevent cell detachment.

### Transwell assay

The indicated cells were resuspended in DMEM without FBS as 1 × 10^5^/ml. A total of 0.2 mL of cell suspension were added to the upper chamber of the transwell coated with matrigel (Corning, Shanghai, China), and the culture medium containing 10% FBS was added to the lower chamber for 24 h. Carefully wipe the cells in upper chamber with a cotton ball, fixed the cells which invaded into the lower chamber with methanol, and stained with 3% crystal violet. The number of transmembrane cells was counted in five fields. For the rescue assay, the cell concentration was 1 × 10^4^/ml. All operations require caution to prevent cell detachment.

### Dual luciferase assay

Majorly, 5000 bladder cancer cells (T24 with or without NOD1 overexpression) were seed into 24-well plates without antibiotics until. Cignal Finder 10-Pathway Reporter Arrays (Qiagen) were employed to identify the potential pathway(s) regulated by NOD1 as our previous reports [43, 44].

To validate NOD1 and MARCH7 effect on NF-κB pathway, NF-κB luciferase reporter which contains 3 NF-κB-binding sites and internal control pRL-TK plasmid, NOD1 and MARCH7 were transfected into T24 as indicated. 48 h later, dual luciferase reporter assay kit (Promega) detected the luciferase activities of the cells following the instruction of manual, with reporter luciferase activity normalized to renilla luciferase activity.

### Immunoprecipitation assay

Immunoprecipitation was employed to detect the interaction between NOD1 and MARCH7, and determine the ubiquitination of HA-NOD1 or NOD1, as described previously [45]. Briefly, HA-NOD1 and Flag-MARCH7 were transfected into the cells. After 48 h of transfection, MG132 (10 μM) was added and incubated for another 24 h. Samples were collected at 2000 rpm for 5 min, suspended in 2% SDS buffer, boiled for 10 min, and sheared with a sonication device. Afterwards, the supernatant was rotated with anti-HA or anti-FLAG for 12 h, followed by protein A- or G-agarose beads for another 2 h. The bead-containing samples were washed thrice with pre-cooled PBS. Finally, the beads were removed by centrifugation at 20,000 ×*g* for 3 min. The residual supernatant was collected and boiled in 5X SDS loading buffer for 10 min. Besides, IgG was used as a negative control. These samples were loaded onto SDS-PAGE gels for immunoblotting with antibodies to determine the indicated proteins and protein ubiquitination.

### In vitro ubiquitination detection

To determine MARCH7 may directly interact with NOD1, the Flag-MARCH7-WT, Flag-MARCH7-Mut, HA-NOD1 proteins were purified by Hangzhou Jingjie Biotechnology Co., Ltd.. Co-IP assay to determine the direct interaction between MARCH7 and NOD1 as described above. The ubiquitination detecting kit (Cat. no. VB2948, Viva Bioscience, Shanghai, China) was applied to examine the ubiquitination of NOD1. Briefly, the purified NOD1 protein was mixed with purified Flag-MARCH7-WT or Flag-MARCH7-Mut, and the mixed proteins were incubated in the tubs containing E1, E2, ATP, ubiquitin for 12 h. Afterwards, they samples were used for Co-IP using anti-HA to determine the effects of MARCH7-WT or -Mut on ubiquitination of HA-NOD1. Finally, these samples were added loading buffer for western blotting analysis.

### Xenograft model

Male BALB/c nude mice aged 4–5 weeks were purchased from GemPharmatech (Chengdu, China) and housed in an SPF room. After 7 days adaptive phase, the mice were blindly and randomly divided as indicated groups (3 mice per group). The procedure for using the animals followed the Guidance Suggestions for the Care and Use of Laboratory Animals formulated by the Ministry of Science and Technology of China, and animal experiments were approved by the Animal Ethics Committee of West China Second University Hospital. The optimized experimental procedures minimized the number of experimental animals and alleviated their suffering. For xenograft tumor generation, 2 × 10^6^ T24 cells and sorted stem cells were injected subcutaneously into the flank. The length and width of the tumors were measured with Vernier calipers every 4 days, and the tumor volume was calculated as length × width^2^/2. When the weight loss of an animal reaches 20–25%, or when the animal exhibits cachexia or wasting symptoms, or when the size of a solid tumor exceeds 10% of the animal’s body weight, the animal's life should be terminated. The nude mice were sacrificed at the indicated endpoint, and the tumors were excised and weighed.

### Statistical analysis

Statistical analyses were performed using SPSS version 19.0. All the data were from three independent experiments. The measurement data are shown as $\overline{X }\pm S$. One-way ANOVA was applied for comparison between multiple groups, and the LSD-t test was performed for pairwise comparison between groups. Enumeration data were analyzed using the *χ*^2^ test. P < 0.05 was considered statistically significant at P < 0.05.

## Results

### Decreased expression of MARCH7 in bladder cancer tissues

**Fig. 1:**
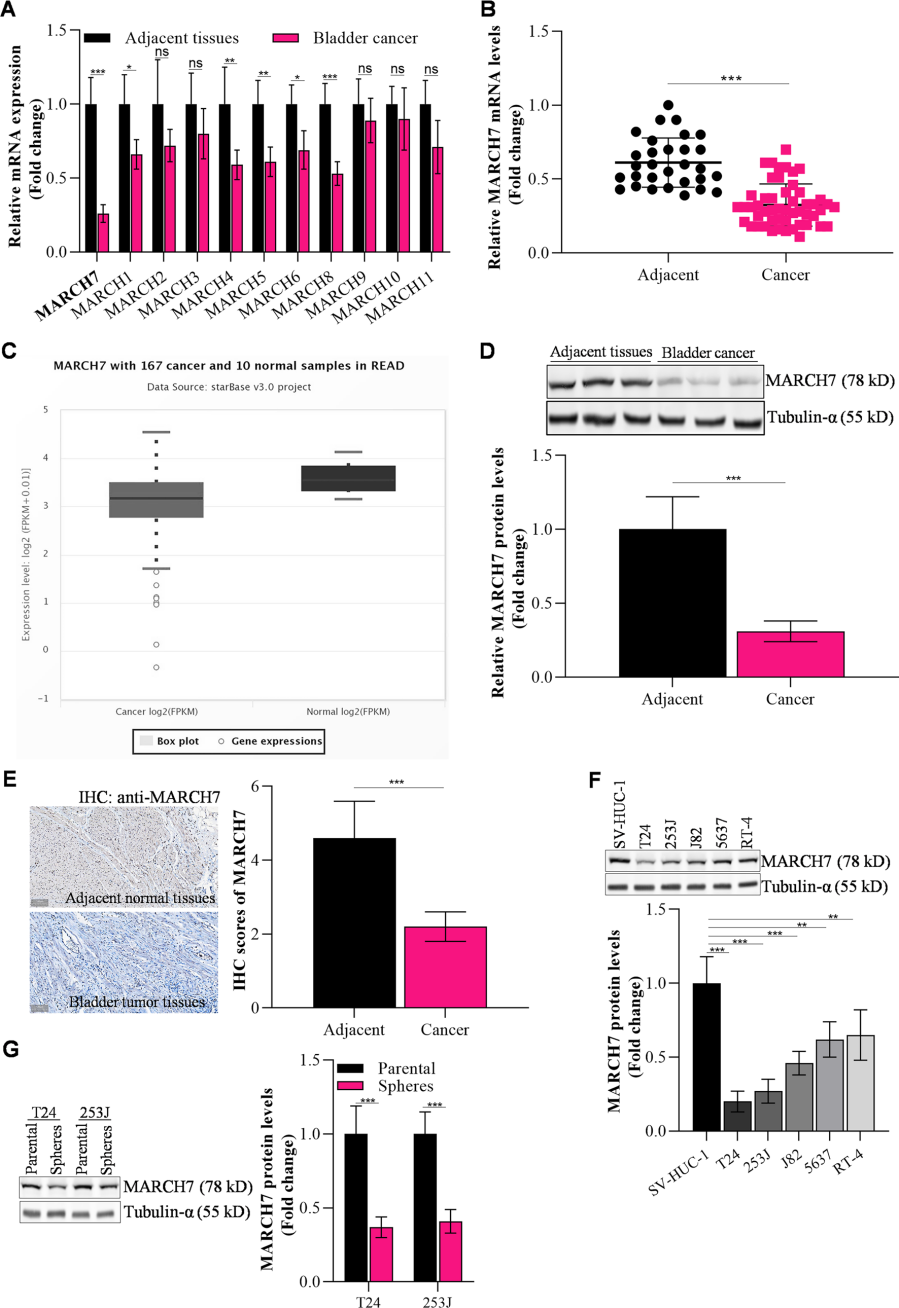
MARCH7 was a suppressor gene in bladder cancer. **A** The levels of MARCH family genes in bladder cancer tissues (N = 5) detected by q-PCR, *p < 0.01 vs. adjacent tissues (N = 5). **B** The level of MARCH7 in bladder cancer tissues (N = 30) detected by q-PCR, *p < 0.01 vs. adjacent tissue (N = 51). **C** Starbase database analysis showed that MARCH7 level was lower in bladder cancer tissues compared to adjacent tissues. **D** The protein level of MARCH7 in bladder cancer tissue (N = 3), *p < 0.01 vs. adjacent tissue (N = 3). **E** IHC examined MARCH7 protein levels in adjacent tissues or bladder cancer tissues (N = 3), *p < 0.01 vs. adjacent tissues. **F** WB analyzed and quantified MARCH7 level in bladder cancer cell lines, *p < 0.01 vs. SV-HUC-1 cells. **G** WB analyzed and quantified MARCH7 expression in T24 and 253 J generated spheres and parental cells, *p < 0.01. Each assay was repeated 3 times independently

The levels of MARCH family genes (MARCH1-11) in bladder cancer tissues were determined using qPCR. Among these genes, MARCH7 showed significantly decreased expression in bladder cancer tissues compared to adjacent tissues (Fig. 1A). This finding was further validated in a larger set of clinical samples, where MARCH7 expression was found to be significantly lower in bladder cancer tissues compared to control normal tissues (Fig. 1B). Consistent with these findings, analysis of the STARBASE database also revealed lower expression of MARCH7 in bladder cancer tissues (Fig. 1C). Additionally, protein analysis through western blot (Fig. 1D) and immunohistochemistry staining (Fig. 1E) confirmed that MARCH7 protein levels were significantly lower in bladder cancer cells compared to adjacent tissue. The analysis of various bladder cancer cell lines (T24, 253 J, J82, 5637, RT-4) and an immortalized bladder epithelial cell line (SV-HUC-1) showed consistent results, with bladder cancer cell lines exhibiting lower MARCH7 expression compared to the control cell line (Fig. 1F). Compared to the parental cells (T24 and 253J), decreased MARCH7 expression was also observed in spheres, which have cancer stem-like cells (CSCs) properties (Interleukin 6 signaling maintains the stem-like properties of bladder cancer stem cells)(Fig. 1G).

### MARCH7 decreases the stem-like capacities

**Fig. 2:**
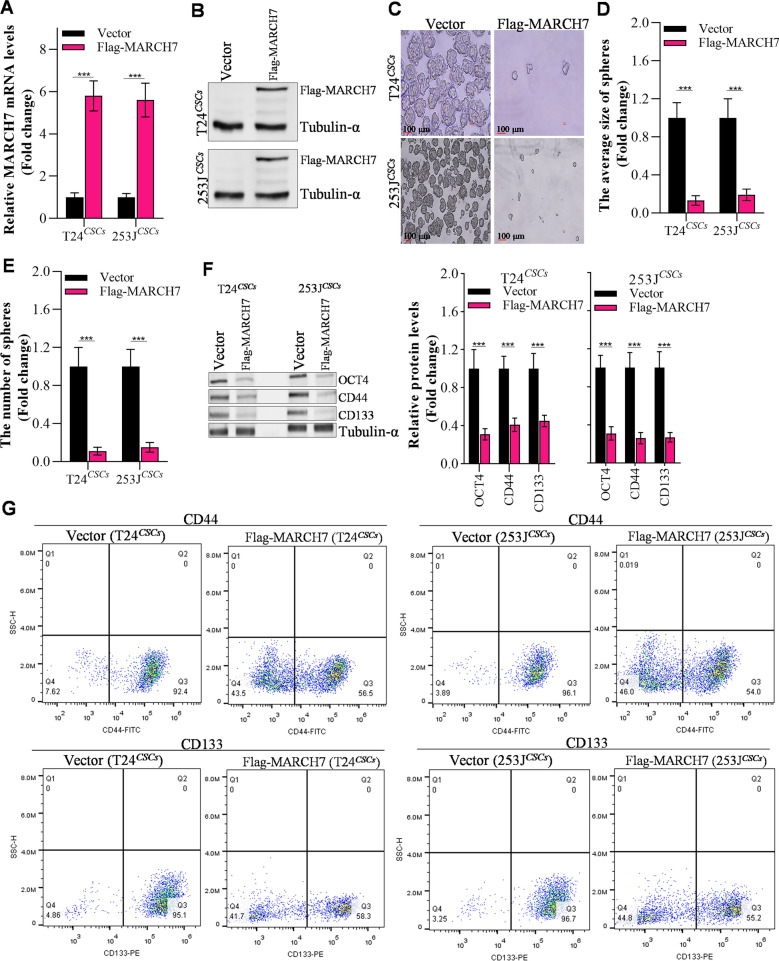
MARCH7 inhibited the stem-like capacities of bladder. **A**, **B** q-PCR and western blot detecting MARCH7 in CSCs (T24^*CSC*^ and 253 J^*CSC*^), *p < 0.01 vs. vector group. **C**–**E** The effect of MARCH7 on the spheroid formation ability and spheroid size, and spheroid number of T24^*CSC*^ and 253J^*CSC*^. **F** The effect MARCH7 on stem-like markers in T24^*CSC*^ and 253J^*CSC*^, *p < 0.01 vs. vector group. **G** Flow cytometry assay detect stem-like markers in T24^*CSC*^ and 253J^*CSC*^ when overexpressed MARCH7. All experiments were conducted in triplicate, and each assay was repeated 3 times independently

To investigate the role of MARCH7 in maintaining bladder cancer stem-like properties, we overexpressed MARCH7 in T24^*CSCs*^ and 253J^*CSCs*^ cells. The overexpression of MARCH7 was confirmed at both the mRNA and protein levels using RT-qPCR and western blot, respectively (Fig. 2A, B). Functional assays revealed that MARCH7 overexpression suppressed the size and number of spheroids formed by T24^*CSCs*^ and 253J^*CSCs*^ cells (Fig. 2C–E). Furthermore, MARCH7 overexpression led to a decrease in the expression of stemness markers, such as OCT4, CD44, and CD133, in bladder cancer stem cells (Fig. 2F). These results indicate that MARCH7 inhibits the stem-like properties of bladder cancer.

### MARCH7 abrogates proliferation and invasion of bladder CSCs

**Fig. 3:**
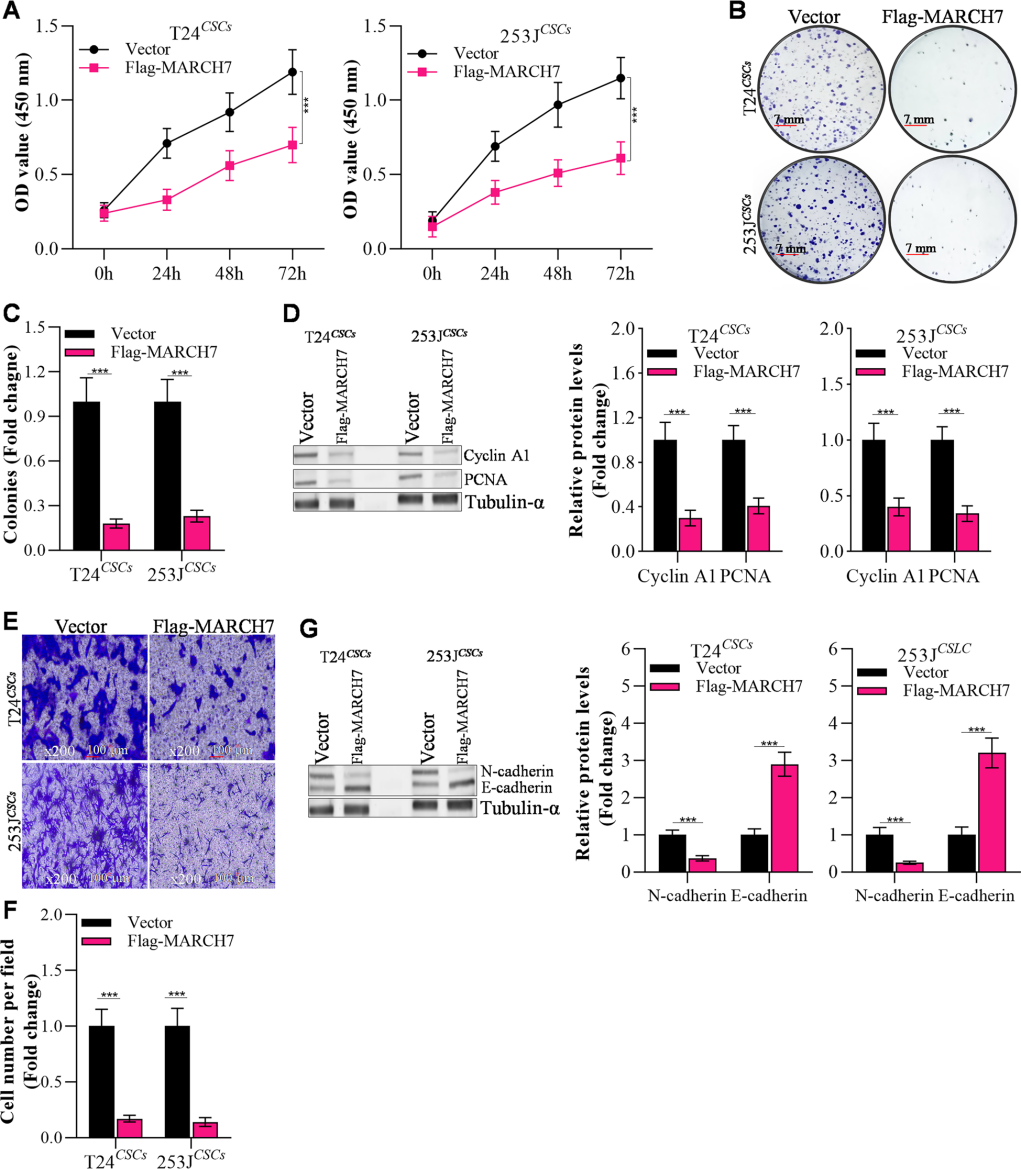
MARCH7 reduces bladder CSCs proliferation, colony formation and invasion. **A** The effects of up-regulated MARCH7 on proliferation of T24 and 253 J derived stem-like cells shown by CCK-8 assay, *p < 0.01 vs. Vector group. **B**, **C** Colony formation in stem-like cells transfected with MARCH7, *p < 0.01 vs. Vector group. **D** MARCH7 modulated proliferation proteins in CSCs, *p < 0.01 vs. Vector group. **E**, **F** Transwell assay detected the effects of MARCH7 on invasion of T24^*CSCs*^ and 253J^*CSCs*^, *p < 0.01 vs. Vector group. **G** Up-regulation of MARCH7 affected invasion genes in T24^*CSC*^ and 253J^*CSC*^, *p < 0.01 vs. Vector group. **D** and **G** The loading control, tublin-α is the same band in Fig. 2F, as the data from the same membrane. All experiments were conducted in triplicate, and each assay was repeated 3 times independently

To assess the impact of MARCH7 on the proliferation and invasion of bladder CSCs, we conducted CCK-8 and colony formation assays. Overexpression of MARCH7 significantly hindered the proliferation of T24^*CSCs*^ and 253 J^*CSCs*^ cells (Fig. 3A–C). Colony formation assays demonstrated a marked reduction in the number of colonies formed by MARCH7-overexpressing cells compared to the control group (Fig. 3C). Additionally, MARCH7 overexpression suppressed the expression of proliferation markers cyclin A1 and PCNA (Fig. 3D). Moreover, MARCH7 overexpression inhibited the invasion ability of T24^*CSCs*^ and 253 J^*CSCs*^ cells, as indicated by the reduced number of invaded cells (Fig. 3E, F). Western blot analysis revealed that MARCH7 overexpression increased the expression of the epithelial marker E-cadherin while downregulating the levels of the mesenchymal marker N-cadherin (Fig. 3G). Collectively, these findings suggest that MARCH7 inhibits the proliferation and invasion abilities of bladder CSCs.

### MARCH7 directly interacts with NOD1

**Fig. 4:**
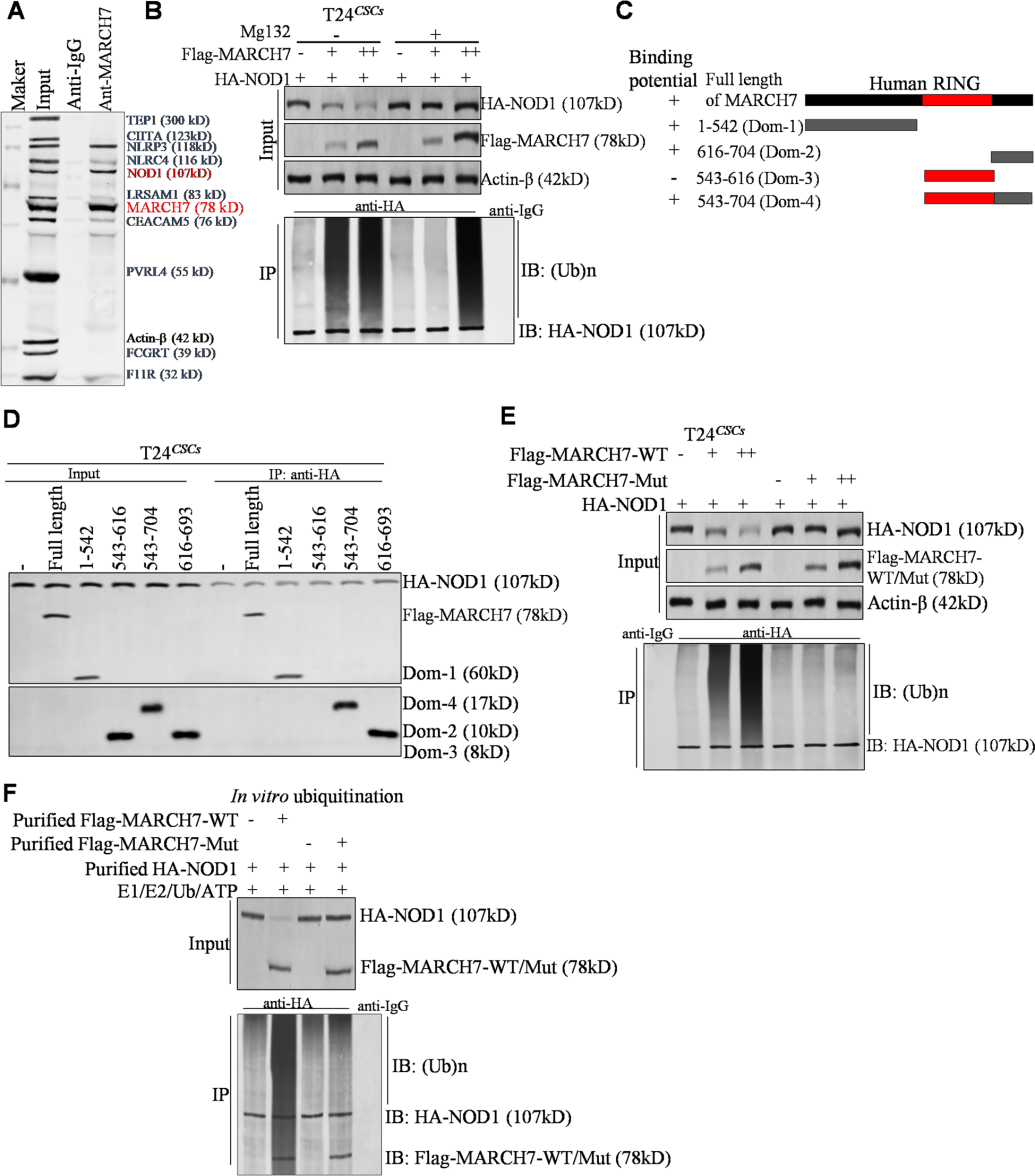
MARCH7 directly targeted NOD1. **A** After co-IP assay, we detected the top 10 UbiBroswer-predicted substrates of MARCH7 using western blot. Here, we studied the interaction between MARCH7 and NOD1 protein. **B** The degradation of HA-NOD1 by Flag-MARCH7 verified by western blot, and the ubiquitination of HA-NOD1 detected by co-IP assay. **C** Schematic representation of the domains of MARCH7. **D** Co-IP detection of each domain of MARCH7 protein interacting with HA-NOD1. **E** Western blot of the degradation of HA-NOD1 by Flag-MARCH7 mutant (Flag-MARCH7-Mut). Co-IP detection showed that Flag-MARCH7-Mut could not induce the ubiquitination-mediated degradation of HA-NOD1. All experiments were conducted in triplicate, and each assay was repeated independently three times. **F** Co-IP and in vitro experiments of ubiquitination determined the effects of MARCH7 on NOD1 ubiquitination by using purified MARCH7 protein and NOD1 protein. All experiments were conducted in triplicate, and each assay was repeated 3 times independently

Using the UbiBrowser tool, potential substrates of MARCH7 were predicted, and a co-immunoprecipitation (Co-IP) assay was performed to identify the top 10 predicted substrates. Among them, NOD1 emerged as the most potential target of MARCH7 (Fig. 4A). To validate NOD1 as a substrate of MARCH7, we treated T24CSC cells with the proteasome inhibitor MG132, which significantly inhibited the ubiquitin–proteasome degradation of NOD1 (Fig. 4B). Conversely, treatment with the lysosome inhibitor E64 had no effect on NOD1 protein levels in T24^CSC^ cells (Additional file 2: Fig. S2), indicating that MARCH7 degrades NOD1 through the ubiquitin–proteasome pathway. Co-IP experiments further elucidated the specific domains of MARCH7 involved in its interaction with NOD1, revealing that Dom1, Dom2, and Dom4 of MARCH7 were required for this interaction, while Dom3 was dispensable (Fig. 4D). To confirm the importance of the E3 ligase activity of MARCH7 in ubiquitin–proteasome degradation, we generated a mutant of MARCH7 (MARCH7-Mut) lacking E3 ligase activity in the Dom3 region. Results showed that MARCH7-Mut failed to induce the ubiquitin–proteasome degradation of NOD1 (Fig. 4E). In vitro ubiquitination and Co-IP assays further demonstrated that MARCH7 directly binds to NOD1, leading to ubiquitination-mediated degradation of NOD1 (Fig. 4F). Additionally, immunohistochemistry staining revealed a significant negative correlation between MARCH7 expression and NOD1 levels in bladder tumors (Additional file 3: Fig. S3). Taken together, these findings indicate that MARCH7 interacts directly with NOD1 and induces the ubiquitination of NOD1 protein through its E3 ligase activity.

### MARCH7 reduces tumorigenicity of CSCs

**Fig. 5:**
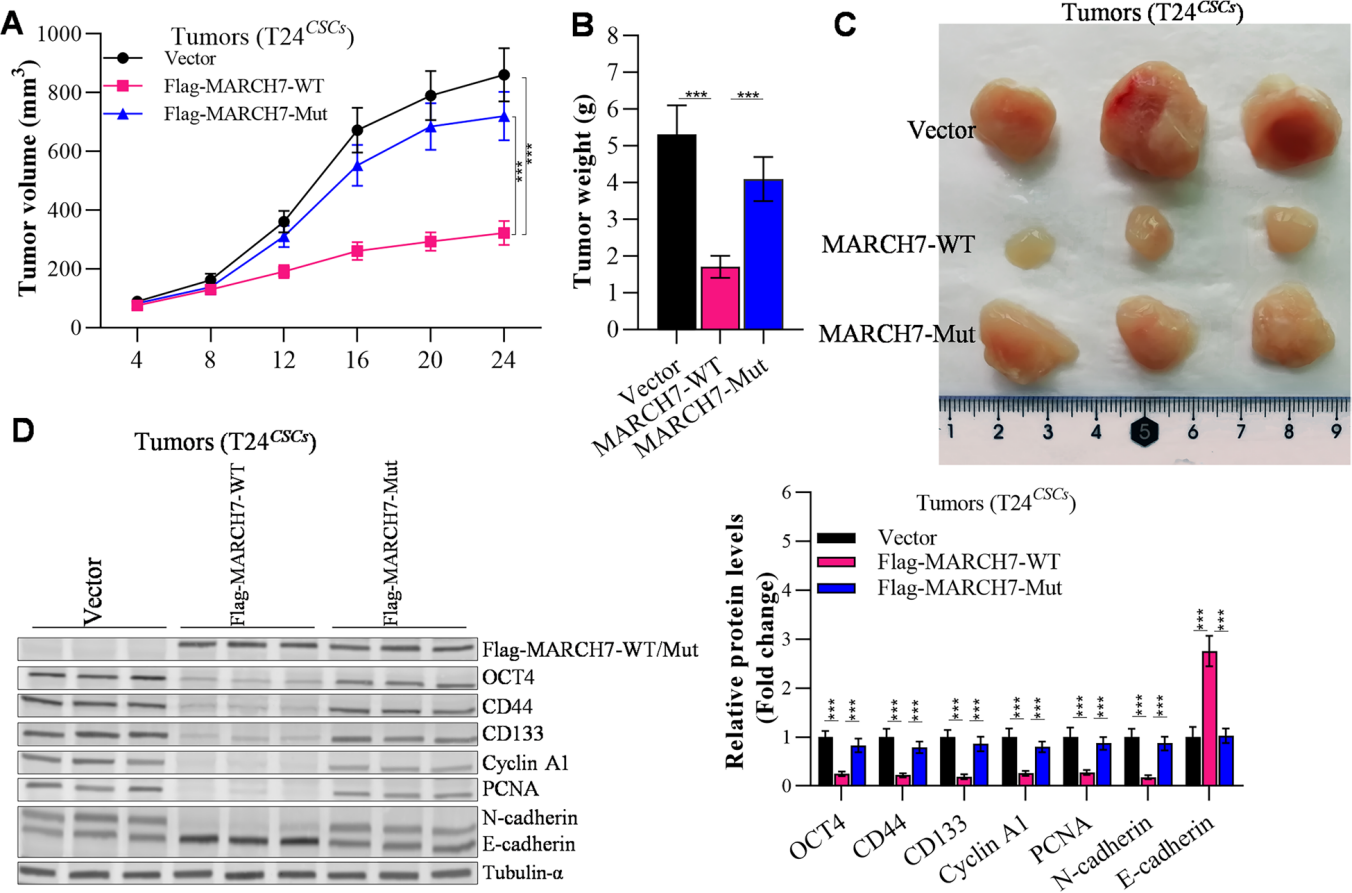
E3 enzyme activity was required for the inhibitory effect of MARCH7 on cancer stem cells evidenced by in vivo model. **A** Volume change of tumor growth. **B** Changes in tumor weight. **C** Photograph of the tumors. **D** Western blot detected the effects of MARCH7 on NOD1 protein levels in the tumors of nude mice. **E** Cancer cell stemness-, proliferation-, invasion- and metastasis-, and apoptosis-related gene expression in xenograft tissues evidenced by q-PCR analysis. Here, *p < 0.01 vs. Vector group, ^#^p < 0.01 vs. MARCH7-WT group. All experiments were conducted in triplicate, and each assay was repeated 3 times independently

To investigate the impact of MARCH7 on tumorigenesis, we established xenograft models using T24^*CSCs*^ cells transfected with the vector, MARCH7-WT, or MARCH7-Mutant. Tumors derived from the MARCH7-overexpressing group exhibited significantly slower growth compared to the control group (Fig. 5A). Furthermore, the tumor volume and weight were significantly lower in the MARCH7 group compared to the vector and MARCH7-Mutant group (Fig. 5A–C). Western blot analysis of xenograft tumors revealed that MARCH7 reduced the expression of OCT4, CD44, CD133, Cyclin A1, PCNA, and N-cadherin, while increasing the expression of E-cadherin (Fig. 5D). In contrast, the MARCH7-Mutant had no significant effect on tumor growth or the expression of the aboved mentioned proteins (Fig. 5). These results suggest that MARCH7 inhibits tumorigenesis and stemness in an E3 ligase-dependent manner.

### MARCH7 alleviates NOD1 induced bladder CSCs properties

**Fig. 6:**
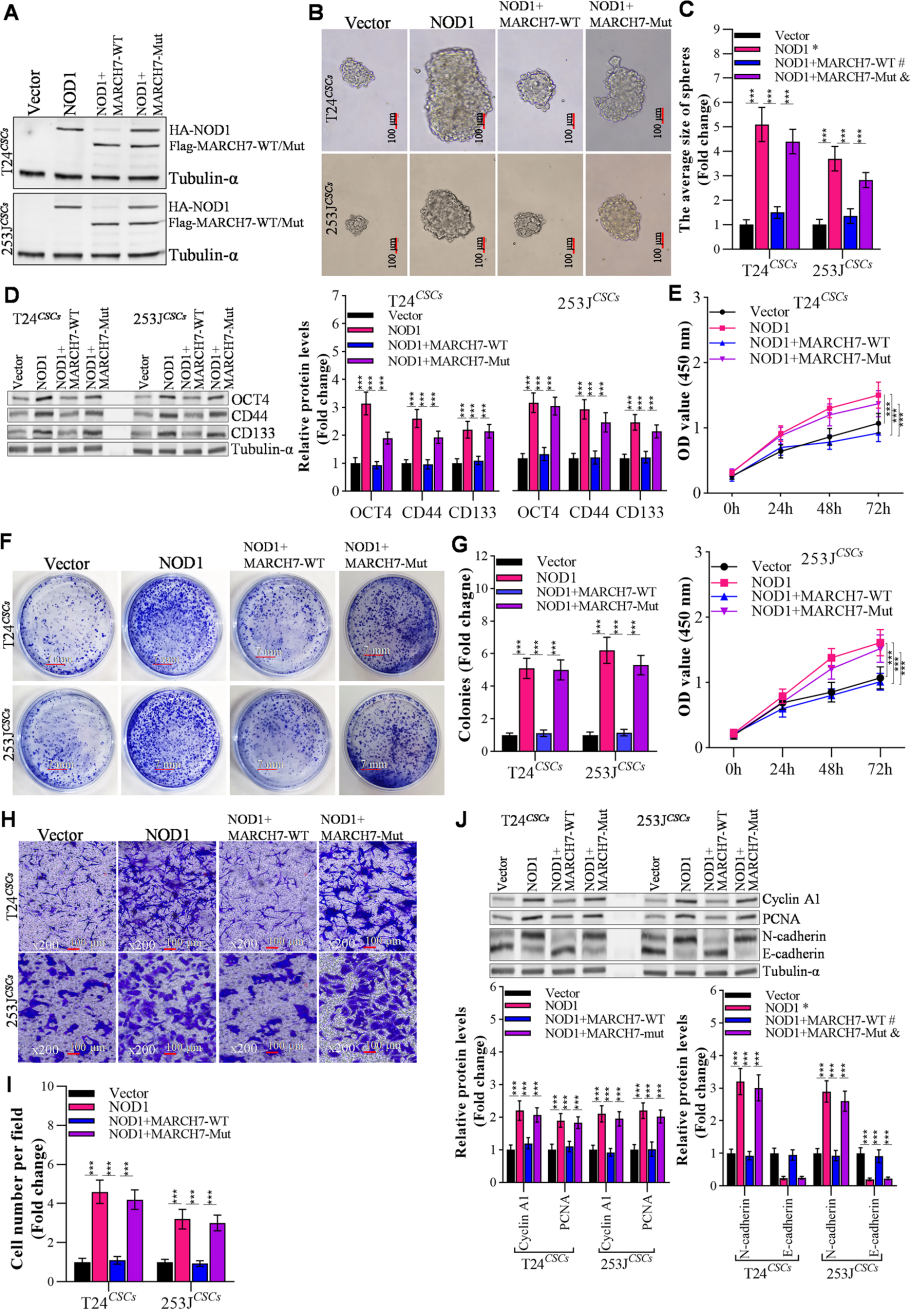
MARCH7 reversed NOD1 induced CSCs stem-like properties. **A** Western blot determined the effects of MARCH7 on the NOD1 protein levels in T24^*CSCs*^ and 253J^*CSCs*^. **B**, **C** Overexpressed MARCH7 reverses the effects of NOD1 on spheroid formation and spheroid size of T24^*CSCs*^ and 253J^*CSCs*^, *p < 0.01 vs. Vector group, ^#^p < 0.01 vs. NOD1 group. **D** MARCH7 reverses the effects of NOD1 on the expression of stem-like markers in T24^*CSCs*^ and 253J^*CSsC*^ detected by immunoblotting, *p < 0.01 vs. Vector group, ^#^p < 0.01 vs. NOD1 group. **E** MARCH7 reverses the effects of NOD1 on the proliferation of T24^*CSCs*^ and 253J^*CSCs*^ detected by CCK-8 assay, *p < 0.01 vs. Vector group, ^#^p < 0.01 vs. NOD1 group. **F**, **G** MARCH7 reverses the effects of NOD1 on colony formation of T24^*CSC*^ and 253J^*CSC*^, *p < 0.01 vs. Vector group, ^#^p < 0.01 vs. NOD1 group. **H** The effect of NOD1 and MARCH7 on the expression of proliferation related proteins in T24^*CSCs*^ and 253J^*CSCs*^, *p < 0.01 vs. Vector group, ^#^p < 0.01 vs. NOD1 group. **I**, **J** Transwell assay detected the effect of NOD1 and MARCH7 on the invasion of T24^*CSCs*^ and 253J^*CSCs*^, *p < 0.01 vs. Vector group, ^#^p < 0.01 vs. NOD1 group. **K** Effects of NOD1 and MARCH7 on the expression of invasion x-related genes in T24^*CSC*^ and 253J^*CSC*^, *p < 0.01 vs. Vector group, ^#^p < 0.01 vs. NOD1 group. **D** and **H** The loading control, tublin-α is the same band, as the data from the same membrane. All experiments were conducted in triplicate, and each assay was repeated 3 times independently

Given the direct interaction between MARCH7 and NOD1, we investigated the roles of the MARCH7/NOD1 axis in bladder cancer cells (Fig. 6A). Overexpression of NOD1 in T24^*CSCs*^ and 253J^*CSCs*^ cells accelerated spheroid formation and increased the size and number of spheroids (Fig. 6B, C), with upregulated stem-like markers OCT4, CD44, and CD133 in bladder CSCs (Fig. 6D). Moreover, CCK-8 assay revealed that upregulated NOD1 significantly enhanced the proliferation of T24^*CSCs*^ and 253 J^*CSCs*^ cells (Fig. 6E). In invasion assay, NOD1 overexpression markedly increased the invasion ability of T24^*CSCs*^ and 253 J^*CSCs*^ cells (Fig. 6F). Co-transfection of MARCH7 and NOD1 effectively rescued the NOD1-induced increase in spheroid formation, proliferation, and invasion (Fig. 6A–F). These findings indicate that MARCH7 can alleviate the maintenance, proliferation, and invasion of bladder CSCs induced by NOD1.

### Reversal of NOD1-induced tumorigenic effects by MARCH7

**Fig. 7:**
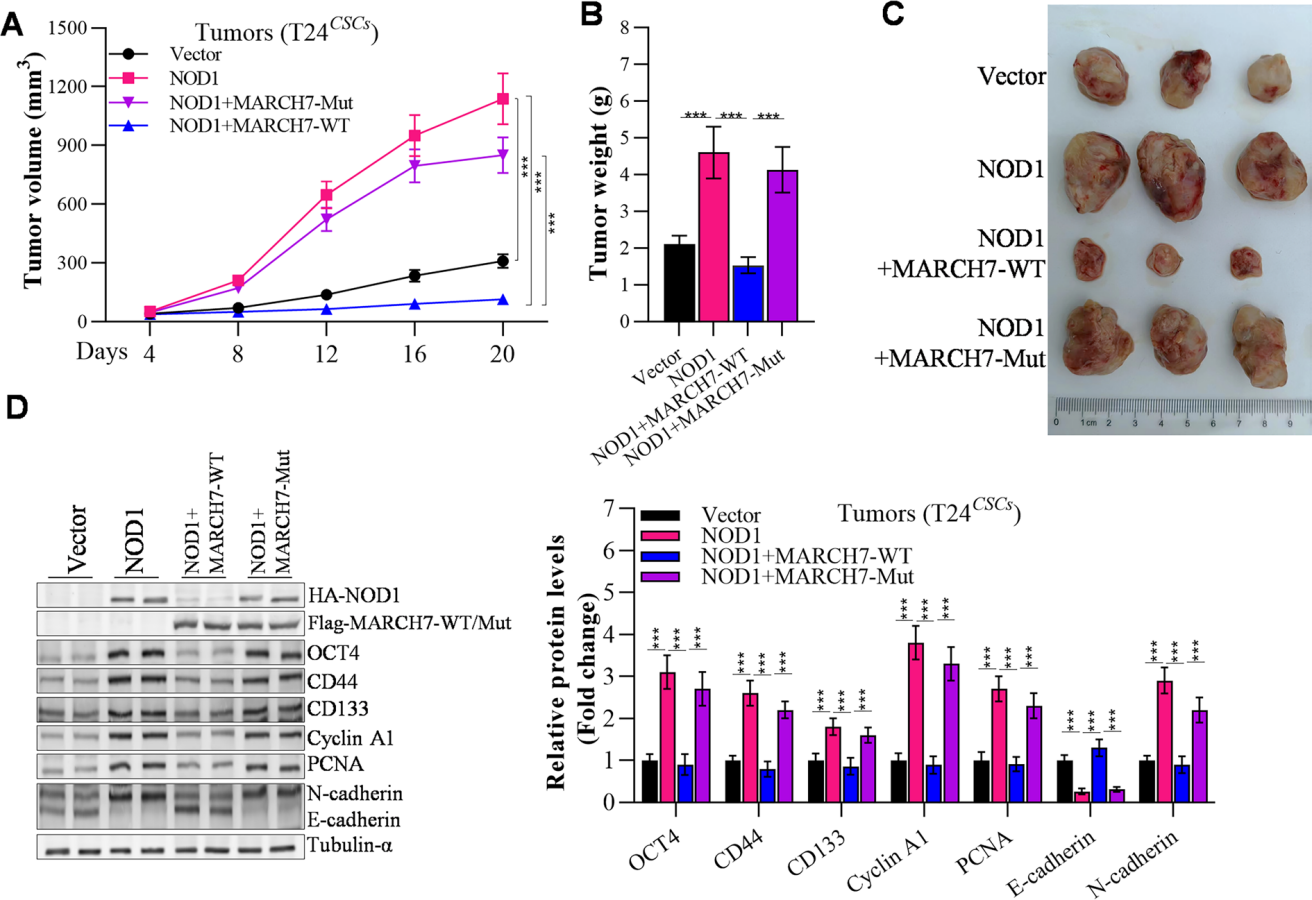
The tumorigenic effect of NOD1 significantly inhibited by MARCH7. **A** Volume change of tumor growth. **B** Changes in tumor weight. **C** Tumor photograph. **D** Western blot determined the effects of MARCH7 on NOD1 protein levels. **E** Expression of cancer cell stemness-, proliferation-, invasion-related genes in xenograft tumors, *p < 0.01 vs. Vector group, ^#^p < 0.01 vs. NOD1 group. All experiments were conducted in triplicate, and each assay was repeated 3 times independently

As depicted in Fig. 7A, the overexpression of NOD1 significantly promoted bladder tumor growth. The NOD1 group exhibited notably higher tumor volume and weight compared to the vector and MARCH7 groups (Fig. 7A–C). Western blot analysis revealed that NOD1 overexpression resulted in increased levels of OCT4, CD44, CD133, Cyclin A1, PCNA, and N-cadherin, while reducing the expression of E-cadherin in the tumors. However, the introduction of MARCH7 partially rescued the dysregulated expression of these proteins induced by NOD1 (Fig. 7D). Notably, the E3 ligase “dead mutation” of MARCH7 failed to reverse the tumorigenic effects of NOD1 in xenograft tumors, as well as the expression of proteins involved in cancer stemness, proliferation, and invasion (Fig. 7A–D).

## Discussion

The incidence of bladder cancer is on the rise, and current treatment strategies show limited efficacy [46]. CSCs have gained considerable attention as a key factor contributing to bladder cancer progression [4]. In this study, we focused on investigating the role of MARCH7 in bladder CSCs. We confirmed that MARCH7 expression was notably lower in bladder cancer tissues and cells, particularly in bladder CSCs. Moreover, overexpression of MARCH7 significantly suppressed the stem-like capacities and biological behavior of bladder cancer stem cells.

Bladder CSCs play critical role in bladder cancer progression and treatment resistance [7, 8]. Here, we isolated bladder CSCs from T23 and 293J cell lines by the ALDEFLUOR^®^ assay (Stemcell Technologies), as previously reported [9]. The CSCs biomarkers, such as CD44 and CD133 were checked by FACS and immunoblotting, and the in vivo and in vitro CSCs properties assays proved that this protocol sorted side population is cancer stem-like cells. However, our finding and data was generated from bladder cancer cell lines which limits the importance of our finding. In our preliminary data, we tried to isolate CSCs form human bladder transitional cell carcinoma with CD44 + and CD133 + magnetic-activated cell separation purification system. However, we failed to generate CSCs population as pollution or limited cell number.

The MARCH family proteins exhibit a relatively conserved structure, characterized by a RING-CH domain followed by zero, two, or more C-terminal transmembrane domains [47]. Among these properties, the RING-CH domain and transmembrane domains are particularly prominent [48]. In ovarian cancer cells, MARCH7 has been shown to upregulate the Wnt/β-catenin pathway, resulting in nuclear translocation and accumulation of β-catenin [49]. In our study, we conducted q-PCR screening and observed the most noticeable change in MARCH7 expression within the Ring E3 family. These findings indicate that MARCH7 contributes to the development of bladder cancer through its E3 enzyme activity. Our study further demonstrated that MARCH7 inhibits the tumorigenesis of cancer stem cells by leveraging its E3 enzyme activity.

Additionally, we established that NOD1 is a substrate of the E3 ligase MARCH7 through co-immunoprecipitation and western blotting. Both in vivo and in vitro models demonstrated that MARCH7 counteracted the effects of NOD1 on proliferation and invasion of bladder CSCs. MARCH7 interacted with NOD1, leading to its ubiquitination and subsequent degradation. Notably, the tumor-promoting effect of NOD1 was significantly mitigated by MARCH7. Intriguingly, while NOD1 and NOD2 often function synergistically in various cellular mechanisms [31, 36, 37], our study revealed that MARCH7 exclusively interacted with NOD1 and not NOD2 in bladder cancer cells (Additional file 4: Fig. S4).

Previous studies have reported conflicting roles for NOD1 in different malignancies [32–35]. Some studies suggest that NOD1 activation primarily contributes to tumor suppression, particularly through the RIP2/TAK1/MAPK pathway-mediated apoptosis of breast tumor growth [32]. In our investigation, we found that NOD1 promoted the stemness of bladder cancer cells, and MARCH7 overexpression abolished the stemness-inducing effects of NOD1. Furthermore, our data indicated that NOD1 activates the NF-κB pathway in bladder cancer cells (Additional file 5: Fig. S5), consistent with previous reports [36]. Collectively, these findings suggest that MARCH7 inhibits bladder CSCs in a NOD1-dependent manner.

Our study demonstrates that MARCH7 effectively suppresses the stem-like capacities of bladder cancer cells by interacting with NOD1. Consequently, MARCH7 and NOD1 have the potential to serve as clinical markers for prognostic evaluation in bladder cancer patients. Furthermore, they may be valuable targets for the development of novel drugs aimed at treating bladder cancer.

### Supplementary Information

**Additional file 1: Figure S1.** Xenograft assay (**A**) and colony formation (**B**) detected bladder cancer stem-like cells tumorigenicity. **C** WB detected OCT4 expression in bladder cancer stem-like and parental cells.

**Additional file 2: Figure S2.** Lysosome inhibitor (E64) treatment has no effect on NOD1 protein level, and proteasome inhibitor (MG132) treatment abrogates NOD1 protein degradation in bladder cancer stem cells.

**Additional file 3: Figure S3.** IHC analyzes MARCH7 and NOD1 protein levels in 17 bladder tumors (**A**). MARCH7 has negative expression with NOD1 in bladder tumors (**B**).

**Additional file 4: Figure S4.** Co-IP checks the interaction of MARCH7 with NOD1/2.

**Additional file 5: Figure S5.** Screening potential downstream signal pathway of NOD1 by Cignal Finder Cancer 10-Pathway Reporter Array in T24 cells (**A**). Dual luciferase assay confirmed NOD1 activate NF-κB signal, and MARCH7 partial blocked this activation (**B**).

## Data Availability

The original data presented in this study are included in the article/additional files, and further inquiries can be directed to the corresponding authors.
