# Supplementary figures and images for: Membrane-associated RING-CH 7 inhibits stem-like capacities of bladder cancer cells by interacting with nucleotide-binding oligomerization domain containing 1
Source: Cell Biosci. 2024 Mar 10;14:32. doi: 10.1186/s13578-024-01210-y (PMC10926635; doi:10.1186/s13578-024-01210-y)

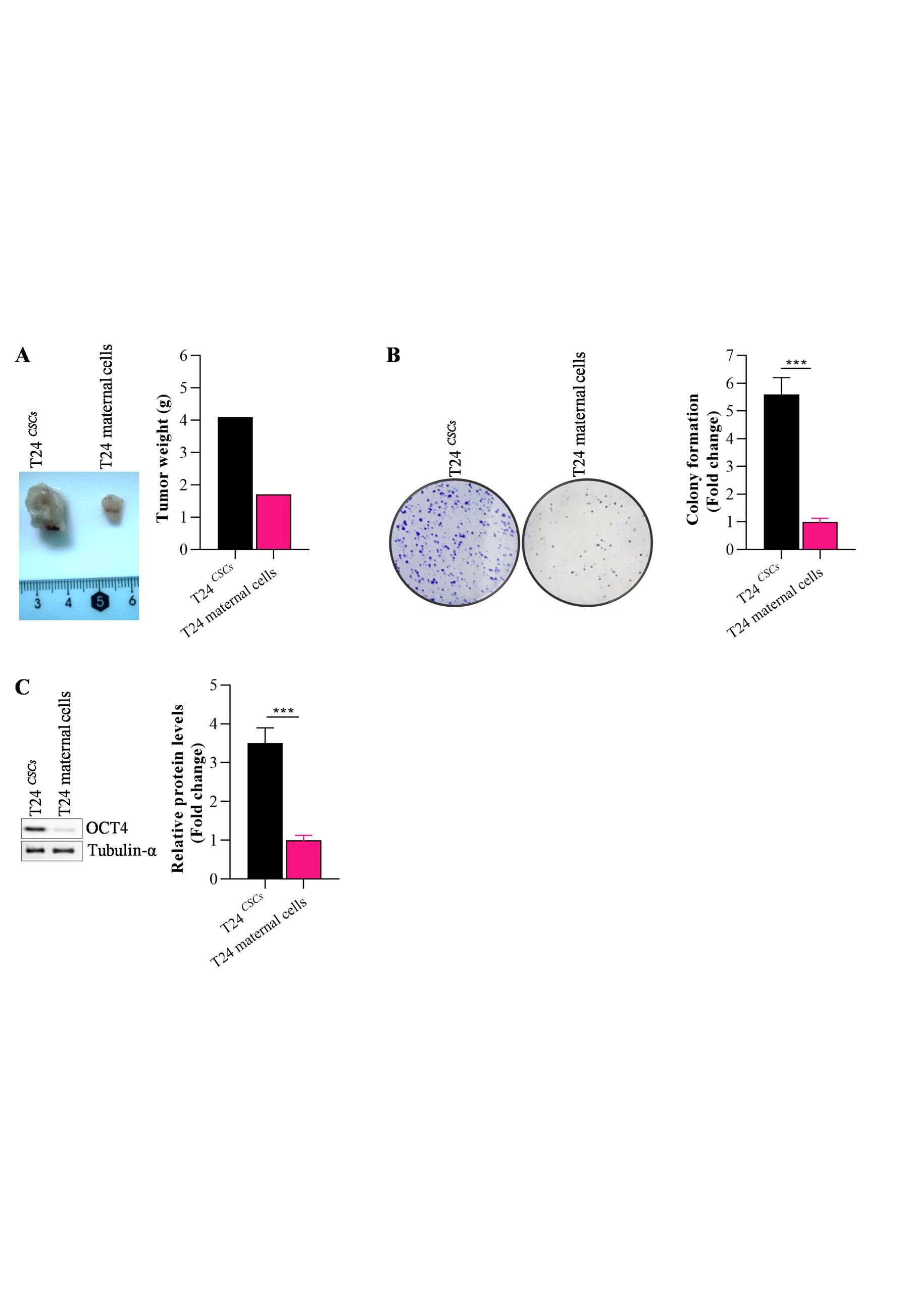

Supplement: Supplementary file 1 — Additional file 1: Figure S1. Xenograft assay (A) and colony formation (B) detected bladder cancer stem-like cells tumorigenicity. C WB detected OCT4 expression in bladder cancer stem-like and parental cells. [file 13578_2024_1210_MOESM1_ESM.tif]

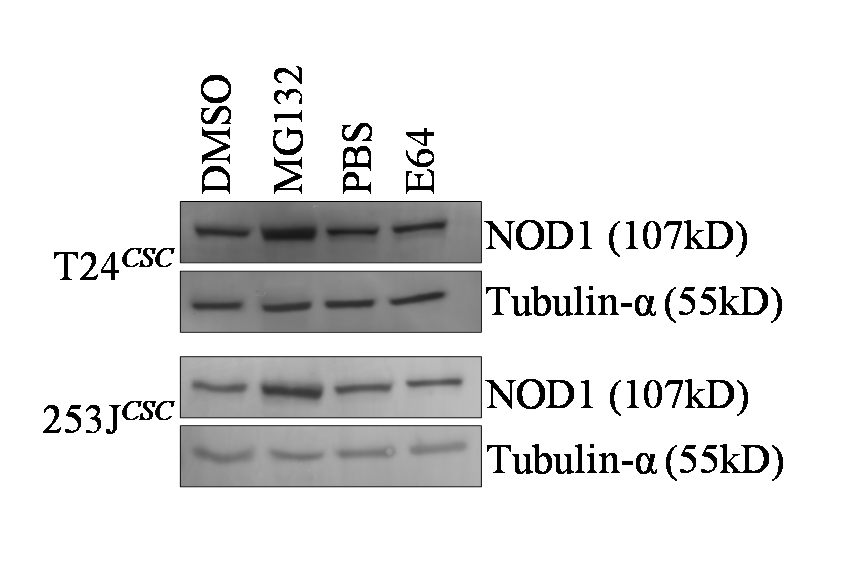

Supplement: Supplementary file 2 — Additional file 2: Figure S2. Lysosome inhibitor (E64) treatment has no effect on NOD1 protein level, and proteasome inhibitor (MG132) treatment abrogates NOD1 protein degradation in bladder cancer stem cells. [file 13578_2024_1210_MOESM2_ESM.tif]

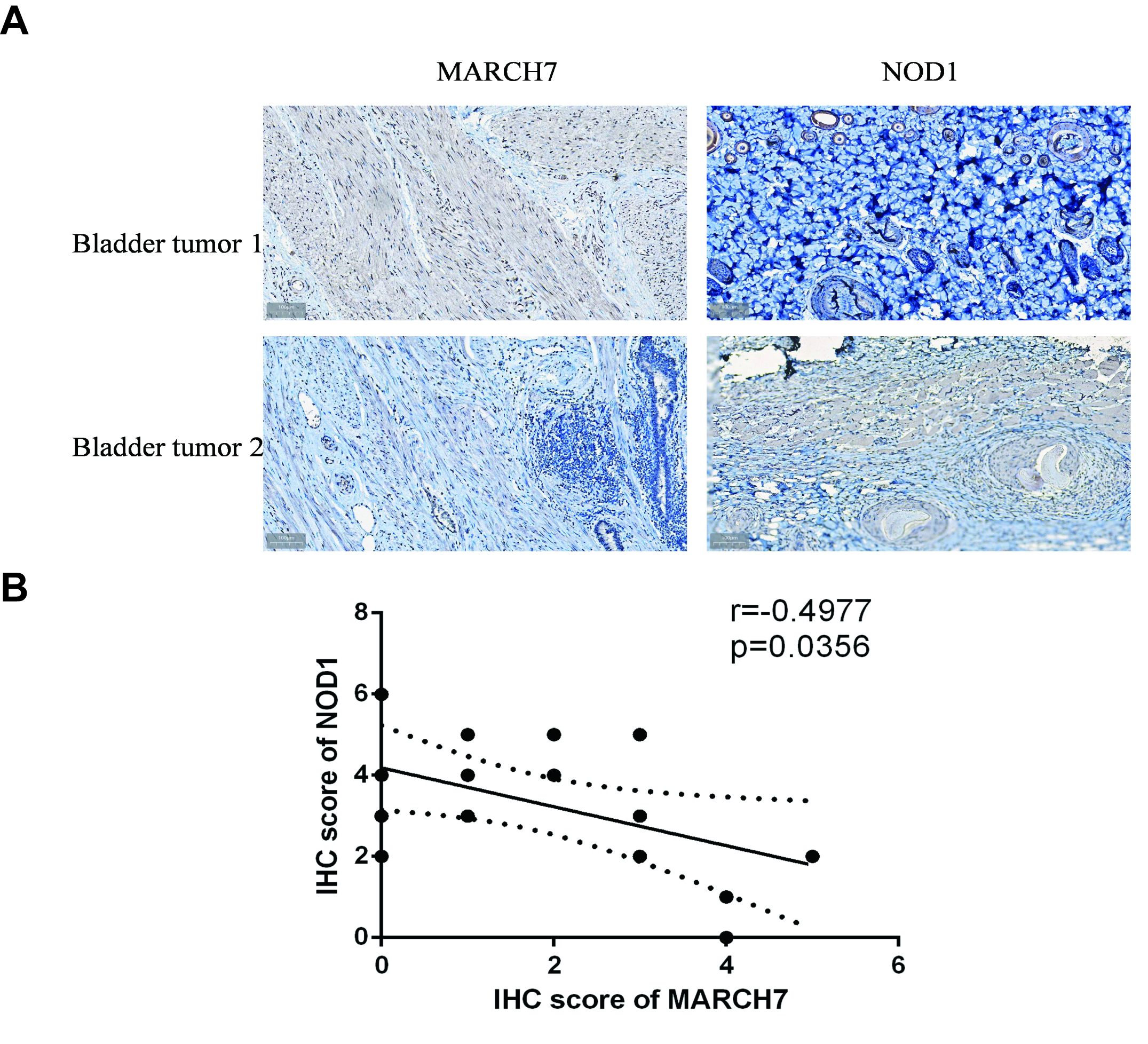

Supplement: Supplementary file 3 — Additional file 3: Figure S3. IHC analyzes MARCH7 and NOD1 protein levels in 17 bladder tumors (A). MARCH7 has negative expression with NOD1 in bladder tumors (B). [file 13578_2024_1210_MOESM3_ESM.tif]

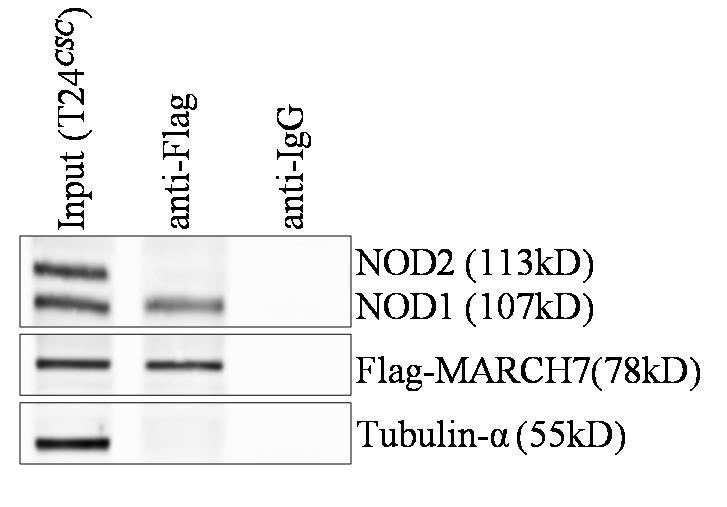

Supplement: Supplementary file 4 — Additional file 4: Figure S4. Co-IP checks the interaction of MARCH7 with NOD1/2. [file 13578_2024_1210_MOESM4_ESM.tif]

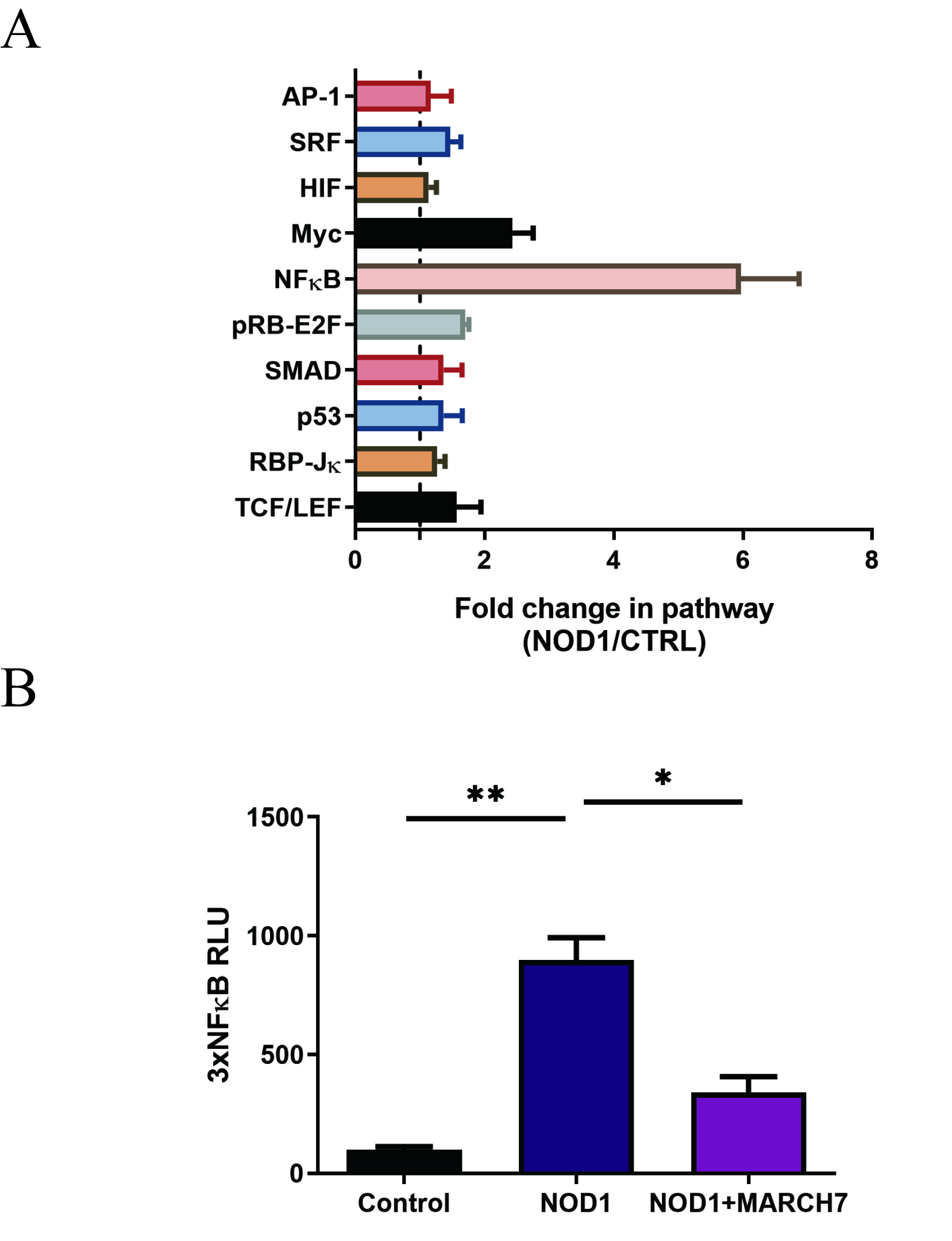

Supplement: Supplementary file 5 — Additional file 5: Figure S5. Screening potential downstream signal pathway of NOD1 by Cignal Finder Cancer 10-Pathway Reporter Array in T24 cells (A). Dual luciferase assay confirmed NOD1 activate NF-κB signal, and MARCH7 partial blocked this activation (B). [file 13578_2024_1210_MOESM5_ESM.tif]
